# BCAS3 exhibits oncogenic properties by promoting CRL4A‐mediated ubiquitination of p53 in breast cancer

**DOI:** 10.1111/cpr.13088

**Published:** 2021-07-09

**Authors:** Zhe Zhou, Rongfang Qiu, Wei Liu, Tianshu Yang, Gen Li, Wei Huang, Xu Teng, Yunkai Yang, Hefen Yu, Yang Yang, Yan Wang

**Affiliations:** ^1^ 2011 Collaborative Innovation Center of Tianjin for Medical Epigenetics Tianjin Key Laboratory of Medical Epigenetics Key Laboratory of Immune Microenvironment and Disease (Ministry of Education) Department of Biochemistry and Molecular Biology School of Basic Medical Sciences Tianjin Medical University Tianjin China; ^2^ Zhejiang Provincial Key Laboratory of Imaging Diagnosis and Minimally Invasive Intervention Research The Fifth Affiliated Hospital of Wenzhou Medical University Lishui Hospital of Zhejiang University Lishui China; ^3^ Beijing Key Laboratory for Tumor Invasion and Metastasis Advanced Innovation Center for Human Brain Protection Department of Biochemistry and Molecular Biology School of Basic Medical Sciences Capital Medical University Beijing China; ^4^ State Key Laboratory of Molecular Oncology National Cancer Center/National Clinical Research Center for Cancer/Cancer Hospital Chinese Academy of Medical Sciences and Peking Union Medical College Beijing China

**Keywords:** breast cancer, breast cancer‐amplified sequence 3, CUL4A‐RING E3 ubiquitin ligase, p53, ubiquitination

## Abstract

**Objectives:**

Breast cancer‐amplified sequence 3 (BCAS3) was initially found to be amplified in human breast cancer (BRCA); however, there has been little consensus on the functions of BCAS3 in breast tumours.

**Materials and methods:**

We analysed BCAS3 expression in BRCA using bio‐information tools. Affinity purification and mass spectrometry were employed to identify BCAS3‐associated proteins. GST pull‐down and ubiquitination assays were performed to analyse the interaction mechanism between BCAS3/p53 and CUL4A‐RING E3 ubiquitin ligase (CRL4A) complex. BCAS3 was knocked down individually or in combination with p53 in MCF‐7 cells to further explore the biological functions of the BCAS3/p53 axis. The clinical values of BCAS3 for BRCA progression were evaluated via semiquantitative immunohistochemistry (IHC) analysis and Cox regression.

**Results:**

We reported that the expression level of BCAS3 in BRCA was higher than that in adjacent normal tissues. High BCAS3 expression promoted growth, inhibited apoptosis and conferred chemoresistance in breast cancer cells. Mechanistically, BCAS3 overexpression fostered BRCA cell growth by interacting with the CRL4A complex and promoting ubiquitination and proteasomal degradation of p53. Furthermore, BCAS3 could regulate cell growth, apoptosis and chemoresistance through a p53‐mediated mechanism. Clinically, BCAS3 overexpression was significantly correlated with a malignant phenotype. Moreover, higher expression of BCAS3 correlates with shorter overall survival (OS) in BRCA.

**Conclusions:**

The functional characterization of BCAS3 offers new insights into the oncogenic properties and chemotherapy resistance in breast cancer.

## Graphical abstract

BCAS3, the WD40 repeat protein, is a new interaction partner of CUL4A‐RING E3 ubiquitin ligase (CRL4A) complex. The cullin subunit of CUL4A acts as a scaffold and is responsible for ROC1 connection to recruit E2, while the other end recruits p53 for ubiquitination degradation through BCAS3 which directly binds to DDB1. The biological process of ubiquitination p53 by BCAS3/CRL4A complex is involved in regulating cell proliferation, apoptosis and chemotherapy resistance in breast cancer.

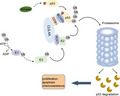

## 1 INTRODUCTION

Breast cancer (BRCA) is the most common malignant tumour in women, mainly divided into four subtypes: ER‐positive, PR‐positive, HER2‐positive and triple‐negative breast cancer (TNBC).^1^ Among breast cancers, approximately 70% are ER‐positive breast cancer.^2^ Clinical data have shown that tamoxifen could decrease the distant recurrence rate and contribute to improving clinical outcome.^3^, ^4^ However, breast cancer is a clinically heterogeneous cancer; it is critical to explore multiple molecular features.

BCAS3 (Breast cancer‐amplified sequence 3), 98% identical to murine *Rudhira*, was originally identified to be amplified and overexpressed in breast cancer.^5^, ^6^ The gene is located on human chromosome 17q23, a region that carries multiple oncogenes and remains amplified in approximately 20% of primary breast carcinomas.^7^ *Rudhira* encodes a predicted WD40 protein and is expressed in angiogenic precursors and embryonic stem (ES) cells. Similarly, previous studies implicated that BCAS3 was also distributed in human ES cells and angiogenesis. These results imply that BCAS3 is involved in the angiogenesis of tumours. In addition, evidence has shown a positive correlation between overexpression of BCAS3 and tumour progression.^8^

Tumour suppressor gene p53 can effectively prevent breast cancer development. Once inactivated, oncogenic activities occur, which can exacerbate metastasis and drug resistance of cancer cells, a hallmark of cancer progression. This is consistent with several previous studies based on mouse experiments and cell‐based assays, indicating that loss of p53 function increases the susceptibility of cells to tumour initiation.^9^, ^10^, ^11^ It is widely known that p53 stability is regulated by post‐translational modifications, including ubiquitination.

The ubiquitin system exerts its major function by targeting substrates for degradation and consequently maintaining cellular homeostasis.^12^ The basic functions of ubiquitination are mainly dependent on the ubiquitin‐activating enzyme (E1), ubiquitin‐conjugating enzyme (E2) and ubiquitin ligase (E3). MDM2 is a crucial E3 ubiquitin ligase that targets p53 for degradation; however, it can be regulated by p53.^13^, ^14^, ^15^ In addition to MDM2, Cullin4A‐RING E3 ubiquitin ligase (CRL4A) could interact with and target p53 for degradation.^16^ At the N terminus of CUL4A, DCAF (DDB1‐CUL4A–associated factors) are formed by binding DDB1 to recruit substrates.^17^ The C terminus attaches ROC1/RBX1 to recruit E2 enzymes, which serve as catalytic centers.^16^ Among additional RING‐type E3 ligases, PIRH2,^18^ CUL1/SKP2,^19^ COP1,^20^ CARP1/2^21^ and CUL5^22^ all ubiquitinate p53 for proteasomal degradation. In addition, various E3 ligases, including HECT‐type (ARFBP and Msl2/WWP1) and U box‐type (CHIP and UBE4B), are also able to elicit p53 degradation.^23^, ^24^, ^25^, ^26^

In this study, we show that the WD40 repeat protein, BCAS3, is a new interaction partner of CRL4A. As a substrate‐specific adaptor, BCAS3 directly interacts with DDB1 to promote p53 polyubiquitination in a CRL4A‐dependent pathway. Functionally, BCAS3 regulates cell proliferation, apoptosis and chemoresistance through the degradation of p53 protein. Overall, these results reveal that BCAS3, an important substrate‐specific adaptor for CRL4A to regulate the p53 stability, acts as a novel prognostic marker and guides chemotherapy regimens for patients with breast cancer.

## 2 MATERIALS AND METHODS

### 2.1 Cell lines

MCF‐7, HEK 293T and MDA‐MB‐231 cell lines were purchased from the Cell Bank of the Chinese Academy of Science (Shanghai, China). The culture medium used for these cells is composed of 89% DMEM, 10% fetal bovine serum and 1% antibiotics.

### 2.2 Cell proliferation and cytotoxicity assay

Cell proliferation and cytotoxicity assay were examined using the Cell Counting Kit‐8 assay (APExBio, USA). Briefly, 1 × 10^3^ cells were seeded in each well of 96‐well plates and incubated for 24 hours. For cytotoxicity assay, cells were incubated for 24 hours after drug treatment, 10 μL CCK‐8 solution was added to each well and incubated for 4 hours, and then, the OD_450_ value was determined.

### 2.3 Colony formation assays

A total of 1 × 10^3^ cells were seeded in each well of 6‐well plates. The cells were placed in an incubator and incubated at 37°C for 14 days. After incubation, the medium was removed and the cells were washed twice with cold PBS. Cells were fixed for 15 minutes with methanol and then stained with 0.5% crystal violet solution for 20 minutes. The relative clone formation rate was analysed as follows: (the number of clones/the number of seeded cells) x 100%.

### 2.4 Apoptosis assay

Apoptosis assays were examined using Annexin V‐FITC/PI‐PE apoptosis kit (Roche, Switzerland). A total of 5 × 10^5^ cells were seeded in each well of 6‐well plates and incubated for 24 hours at 37°C and doxorubicin (1 µmol/L) was added to each well for 24 hours. The cells were trypsinized, centrifuged and made into cell suspensions with 400 μL binding buffer. After staining and incubation for 15 minutes, the cell suspensions were analysed using a FACSCalibur flow cytometer (BD Biosciences, USA) in 1 hour.

### 2.5 Immunopurification‐mass spectrometry

HEK 293T cells were transfected with FLAG‐BCAS3 plasmid for 48 hours and lysed for cellular extracts. The anti‐FLAG M2 affinity gel (Sigma‐Aldrich, USA) was added to the cellular extracts for binding. The gel was washed using cold lysis buffer, and then, FLAG peptide (Sigma‐Aldrich, USA) at a concentration of 0.2 mg/mL was applied to the gel to elute the FLAG‐tagged protein complex. Samples were collected and run at 100 V on Nu‐PAGE 4%‐12% Bis‐Tris gel (Invitrogen, USA). Gels were silver‐stained using Pierce Silver Stain Kit (Thermo Scientific, USA) according to the manufacturer's instructions. The gels with target bands were cut and subjected to LC‐MS/MS sequencing.

### 2.6 Immunoprecipitation and Western blot

Cells were collected after washing with cold PBS and lysed with cold lysis buffer for 30 minutes at 4°C. The cell lysates were incubated with specific antibodies or normal IgG overnight at 4°C. Afterwards, protein A/G Sepharose beads were added and incubated for 2 hours at 4°C. Beads were washed three times with lysis buffer, followed by denatured in 5 x loading buffer at 95°C for 5 minutes. The immunocomplexes were subjected to SDS‐PAGE and immunoblotting. Finally, enhanced chemiluminescence (ECL System, Thermo Scientific, USA) was used for immunodetection. The primary antibodies used are listed in Table S2.

### 2.7 Glutathione S‐transferase (GST) pull‐down experiments

Plasmid expressing GST fusion proteins or GST control were transformed into *E coli* BL21, followed by supplemented with 1 mmol/L IPTG to induce protein expression. Bacterial bodies were ultrasonicated at 40% power to harvest the lysate. Glutathione‐Sepharose 4B beads were added to the supernatant, and the mixtures were incubated for 30 minutes at 4°C. The in vitro‐translated proteins were prepared using the TNT transcription/translation system (Promega, USA). The GST fusion proteins were mixed with the in vitro‐transcribed/translated products and incubated in binding buffer for 2 hours at 4°C. The beads were washed five times with binding buffer and denatured in 2 × loading buffer at 95°C for 5 minutes. Protein bands were detected by Western blot using specific antibodies (Table S2).

### 2.8 In vivo ubiquitination assay

MCF‐7 cells were transfected with BCAS3 shRNA or Control shRNA, followed by treatment with MG132 (20 µmol/L) for 6 hours before harvesting. Briefly, cells were washed in PBS and lysed in lysis buffer supplemented with a 1 × protease inhibitor cocktail. Specific antibodies were added into cellular extracts for incubation overnight at 4°C. Afterwards, protein A/G Sepharose beads were added and incubated for 2 hours at 4°C. Beads were washed three times with lysis buffer, followed by denatured in 5 × loading buffer at 95°C for 5 minutes. The proteins were subjected to SDS‐PAGE and immunoblotting with an anti‐ubiquitin antibody to examine p53 ubiquitination.

### 2.9 In vitro ubiquitination assay

The ubiquitin conjugation reaction buffer kit (Boston Biochem, USA) was used for in vitro ubiquitination assay. CUL4A antibody was used to immunoprecipitate immunocomplexes from MCF‐7 cells. Afterwards, the CUL4A immunocomplex was mixed with bacterially expressed GST‐BCAS3, GST‐p53 in 20 µL ubiquitination reaction containing the following reagents: 2 µL reaction buffer, 1 mmol/L Mg‐ATP solution, 200 µmol/L Flag‐ubiquitin, 50 nmol/L E1 and 500 nmol/L E2 (UbcH5a). Reactions were incubated at 37°C for 60 minutes, followed by denatured in 5 × loading buffer at 95°C for 5 minutes. The lysates were subjected to SDS‐PAGE and immunoblotting.

### 2.10 Statistical analysis

Data were analysed using GraphPad Prism 5 (GraphPad Software Inc) and SPSS 18.0 (SPSS software Inc) and were presented as mean ± SD. A chi‐square test was used to analyse the correlation between BCAS3 expression and clinicopathological parameters. Student's unpaired *t* test was used to analyse differences between the two groups. Spearman's and Pearson's correlation coefficient were used to analyse correlations between groups. Kaplan‐Meier analysis was used to calculate the cumulative survival time. Univariate and multivariate analyses based on the Cox regression model were conducted. *P*‐values <.05 were considered statistically significant.

## 3 RESULTS

### 3.1 BCAS3 is highly expressed in BRCA tissues, and a high level of BCAS3 positively correlates with unfavourable prognosis in BRCA cases

**FIGURE 1:**
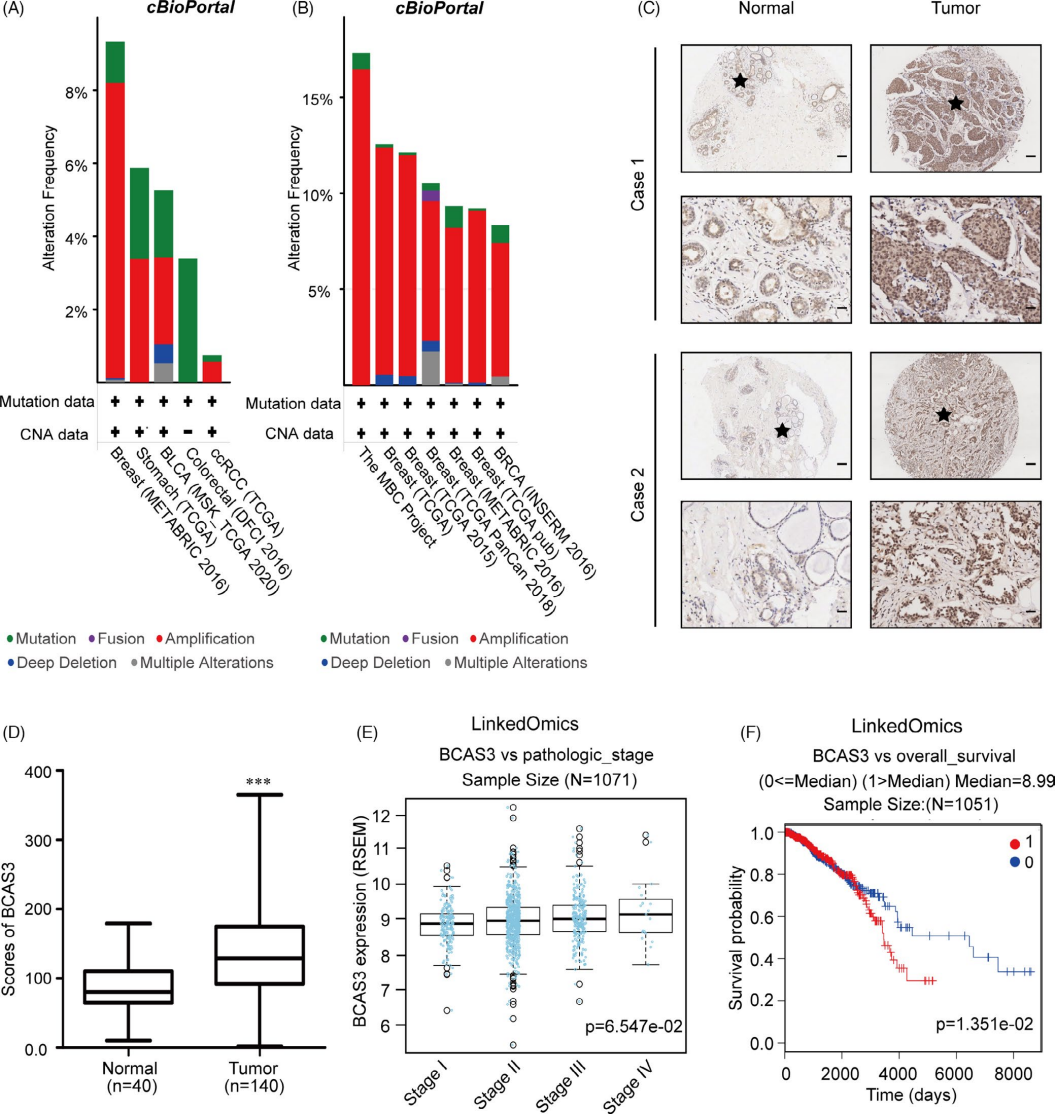
BCAS3 expression is elevated in breast cancer tissues and predicts poor prognosis. (A). Analysis of BCAS3 alteration frequency in the multi‐cancer panel. (B). Analysis of BCAS3 alteration frequency in multiple BRCA cohorts. (C). Representative 2 BRCA cases with high BCAS3 expression in tumour tissue analysed by IHC staining. Scale Bars, 100 µm for ‘up’ and 25 µm for ‘down’. (D). Histoscores for BCAS3 in normal and tumour tissues. (E). BCAS3 mRNA expression is correlated with TNM stages in BRCA samples from LinkedOmics. (F). High BCAS3 expression is correlated with low overall survival in patients with breast carcinoma

To determine BCAS3 expression in human malignancies, we conducted a multi‐cancer analysis of BCAS3 in The Cancer Genome Atlas (TCGA) multi‐cancer panel downloaded from the website of cBioPortal (http://www.cbioportal.org). The results revealed that the BCAS3 amplification frequency was higher in breast cancer than the other four tissue‐derived tumours (Figure 1A). We then detected BCAS3 amplification frequency in six breast cancer cohorts and found that the most frequent BCAS3 alteration was amplification (Figure 1B). Next, we explored the protein level of BCAS3 in human breast carcinoma tissues using IHC staining in 140 cases of breast tumour tissues, and the results indicated an increased level of BCAS3 in tumours compared with normal tissues (Figure 1C,D). To depict the relationship between BCAS3 mRNA expression and disease stages, we performed an analysis of 1071 BRCA samples with BCAS3 expression data from LinkedOmics (http://www.linkedomics.org/admin.php) and demonstrated that BCAS3 mRNA tended to increase with disease progression (Figure 1E). Kaplan‐Meier survival curves showed that cases with elevated BCAS3 expression exhibited decreased overall survival (OS) compared with patients with low BCAS3 expression (*P* < .05) (Figure 1F).

### 3.2 High BCAS3 expression promotes growth, inhibits apoptosis and confers chemoresistance to MCF‐7 cells

**FIGURE 2:**
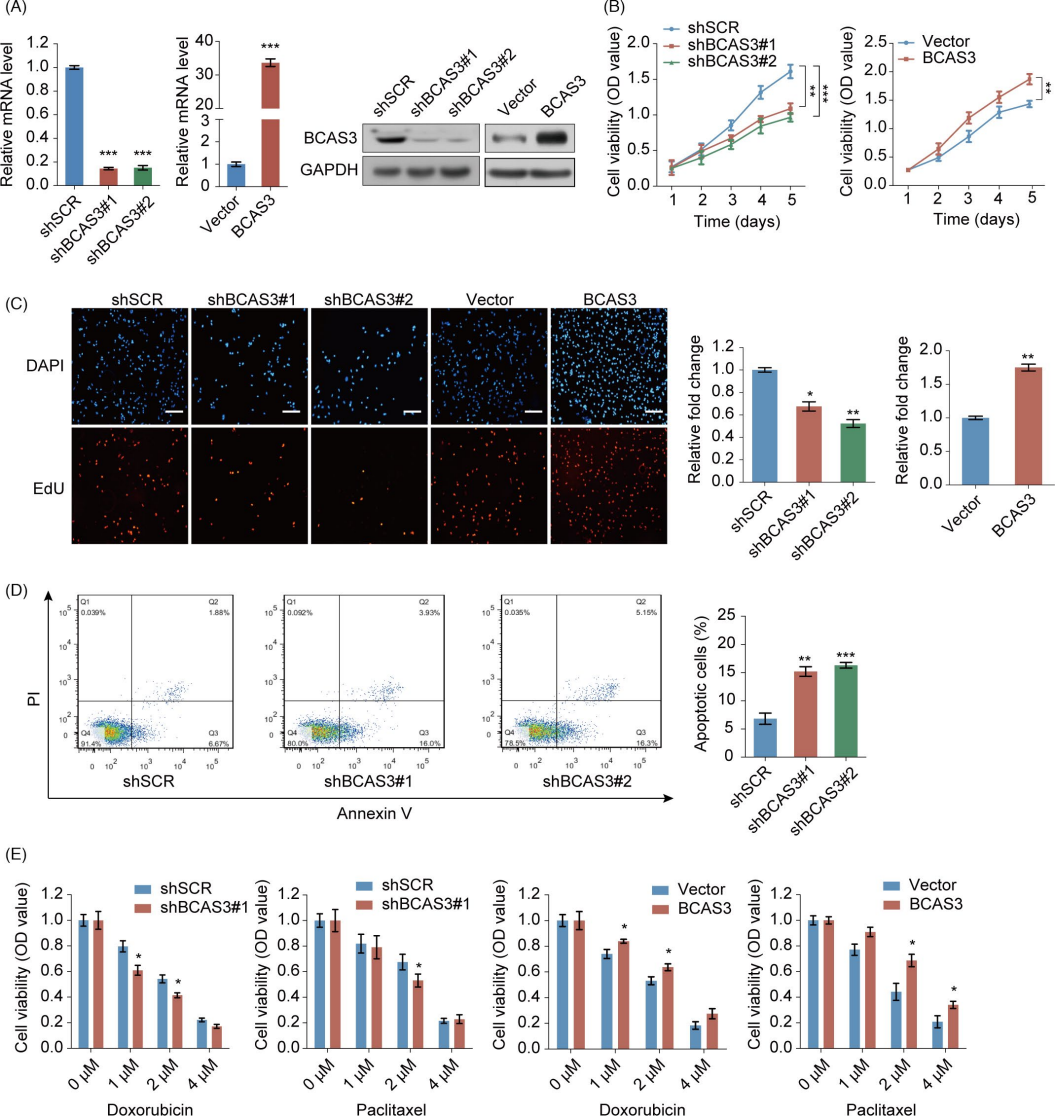
The function of the BCAS3 gene in the regulation of breast tumour cell proliferation, apoptosis and chemoresistance in vitro. (A). Western blot assay and RT‐qPCR are used to examine knockdown and overexpression of BCAS3 in MCF‐7 cells. GAPDH serves as the control. (B and C). CCK‐8 assays (B) and Edu assays (C) are carried out in MCF‐7 stable cell lines over‐expressing or knocking down BCAS3. Scale Bars, 50 µm. (D). Flow cytometry analysis is performed to examine regulation of BCAS3 on cell apoptosis. (E). Cell viability assays are carried out to detect the regulation of BCAS3 on chemoresistance in MCF‐7 cells over‐expressing or knocking down BCAS3. *P < .05; **P < .01; ***P < .001

The above‐mentioned observations prompted us to explore the biological properties of BCAS3 in breast tumorigenesis. First, we constructed stable knockdown and overexpression of BCAS3 in the MCF‐7 cell line. The expression level of BCAS3 was further verified through Western blot and RT‐qPCR (Figure 2A). Next, the regulatory role of BCAS3 on cellular proliferation was carried out using CCK‐8 and Edu assays. BCAS3 knockdown inhibited proliferation, whereas elevated BCAS3 contributed to the growth of MCF‐7 cells (Figure 2B,C). Moreover, we applied flow cytometry analysis to determine the modification of BCAS3 on cell apoptosis. BCAS3 knockdown exerted a greater positive effect on the percentage of early apoptotic cells (Figure 2D). These data suggest that BCAS3 may function in BRCA tumorigenesis.

We then determined whether BCAS3 affected chemoresistance in MCF‐7 cells by gain of function and loss of function. Cells were treated with paclitaxel and doxorubicin at different amounts of drugs for 24 hours, and cytotoxicity was examined through the CCK‐8 assay. We found that BCAS3‐depleted MCF‐7 cells showed a lower survival rate, whereas BCAS3‐overexpressed MCF‐7 cells showed higher survival rates after treatment with paclitaxel for 24 hours at concentrations of 1, 2 and 4 µmol/L. Similar results were showed in the assays for doxorubicin (Figure 2E). Together, these results indicate that BCAS3 overexpression makes MCF‐7 cells gain more resistance to chemotherapy.

### 3.3 BCAS3 interacts directly with CRL4A complex and p53

**FIGURE 3:**
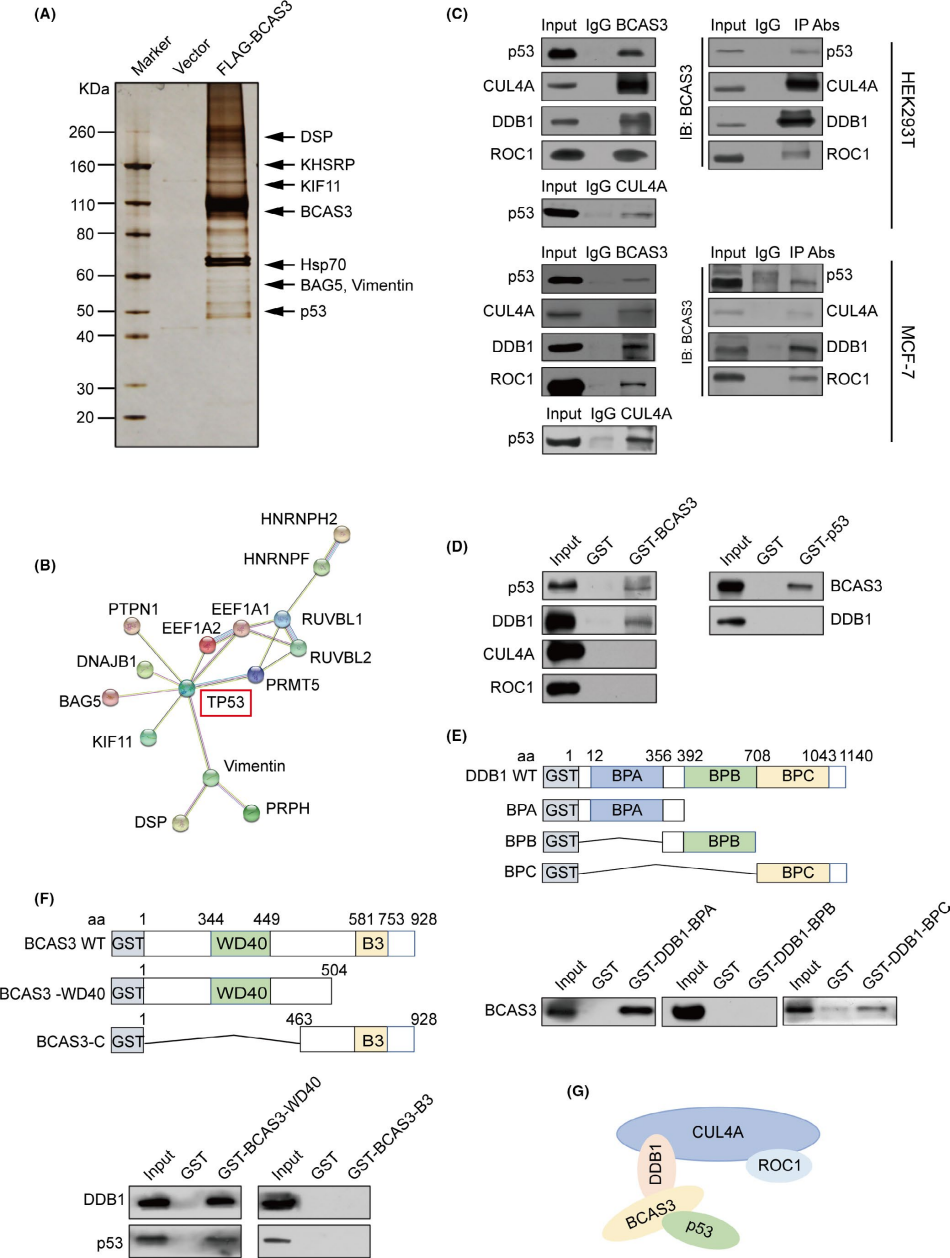
BCAS3 is physically associated with CRL4A complex and p53. (A). BCAS3 physically interacts with p53 in vivo. Cellular lysates are immunoprecipitated with anti‐FLAG. FLAG peptide is applied to elute the FLAG‐tagged proteins. Proteins are fractioned, stained and sequenced by LC‐MS/MS. (B). PPI analysis of BCAS3‐associated proteins. (C). Association of BCAS3 with p53 and CRL4A complex in HEK 293T and MCF‐7 cells. Whole‐cell lysates are immunoprecipitated with specific antibodies and then immunoblotted with indicated antibodies. (D). Co‐incubate the GST fusion protein and the transcribed‐translated protein in vitro for GST pull‐down. (E). BCAS3 directly binds to the BPA domain of DDB1 in vitro. Co‐incubate the GST fusion constructs of DDB1 and the transcribed‐translated BCAS3 protein in vitro for GST pull‐down. (F). WD40 domain of BCAS3 is responsible for interaction with DDB1 and p53. GST pull‐down assays are conducted using constructs of BCAS3 and in vitro‐translated DDB1 and p53 proteins. (G). Schematic diagram of molecular basis between BCAS3, p53 and CRL4A complex

We performed affinity purification‐mass spectrometric assays to identify BCAS3‐associated proteins to define the mechanistic role of BCAS3 in oncogenic properties. FLAG‐tagged vector and FLAG‐tagged BCAS3 were stably transfected in HEK 293T cells. Extracts from cells were purified using the anti‐FLAG affinity gel. Results of mass spectrometric analysis demonstrated that BCAS3 interacted with DSP, KHSRP, KIF11, HSP70, BAG5, vimentin and p53. (Figure 3A). The functional interactions among these identified proteins were shown through a PPI network analysis using the STRING (https://string‐db.org/cgi/input.pl; Figure 3B). As literature reported that the activation of CUL4A led to degradation of p53,^16^ the substrate recruiting of CUL4A‐RING E3 ubiquitin ligases imply that DDB1, which either directly interacts^27^, ^28^ or binds to the substrate protein through the association with WD40 repeat adaptor proteins.^29^, ^30^, ^31^ BCAS3 has a WD40 domain, we explored whether there is an interaction between BCAS3 and CRL4A complex. The interactions between the CRL4A complex and p53 were further confirmed by Western blot analysis in both MCF‐7 cells and 293T cells (Figure 3C), demonstrating that BCAS3 is associated with the CRL4A complex as well as p53 in vivo. The detailed results of the protein mass spectrometric analysis are presented in Table S1.

To explore the molecular mechanism for the interaction between BCAS3 and the p53/CRL4A complex, we conducted GST pull‐down assays using GST‐fused BCAS3 constructs and in vitro‐transcribed/translated p53, DDB1, CUL4A and ROC1. The results revealed that BCAS3 interacts directly with p53 and DDB1, and no interactions were observed between BCAS3 and CUL4A. Data from reciprocal GST pull‐down assays verified the above results (Figure 3D). Next, to further identify the interacting domains between BCAS3 and the CRL4A/ p53, we conducted GST pull‐down assays with GST‐fused BPA, BPB and BPC domains of DDB1.^32^ The results revealed that the BPA domain of DDB1 interacted with BCAS3 (Figure 3E). Subsequently, we constructed the GST‐fused BCAS3‐WD40 domain (1‐504 aa, named BCAS3‐WD40) and the remaining region (463‐928 aa, named BCAS3‐B3), and incubated with in vitro‐transcribed/translated CUL4A, DDB1, ROC1 and p53. The results revealed that the WD40 domain of BCAS3 played a role in the binding to p53 and DDB1 (Figure 3F). Together, these data reveal the physical interaction and detailed molecular basis between BCAS3 and the p53/CRL4A complex (Figure 3G).

### 3.4 BCAS3 promotes p53 protein ubiquitination through a CRL4A‐dependent pathway

**FIGURE 4:**
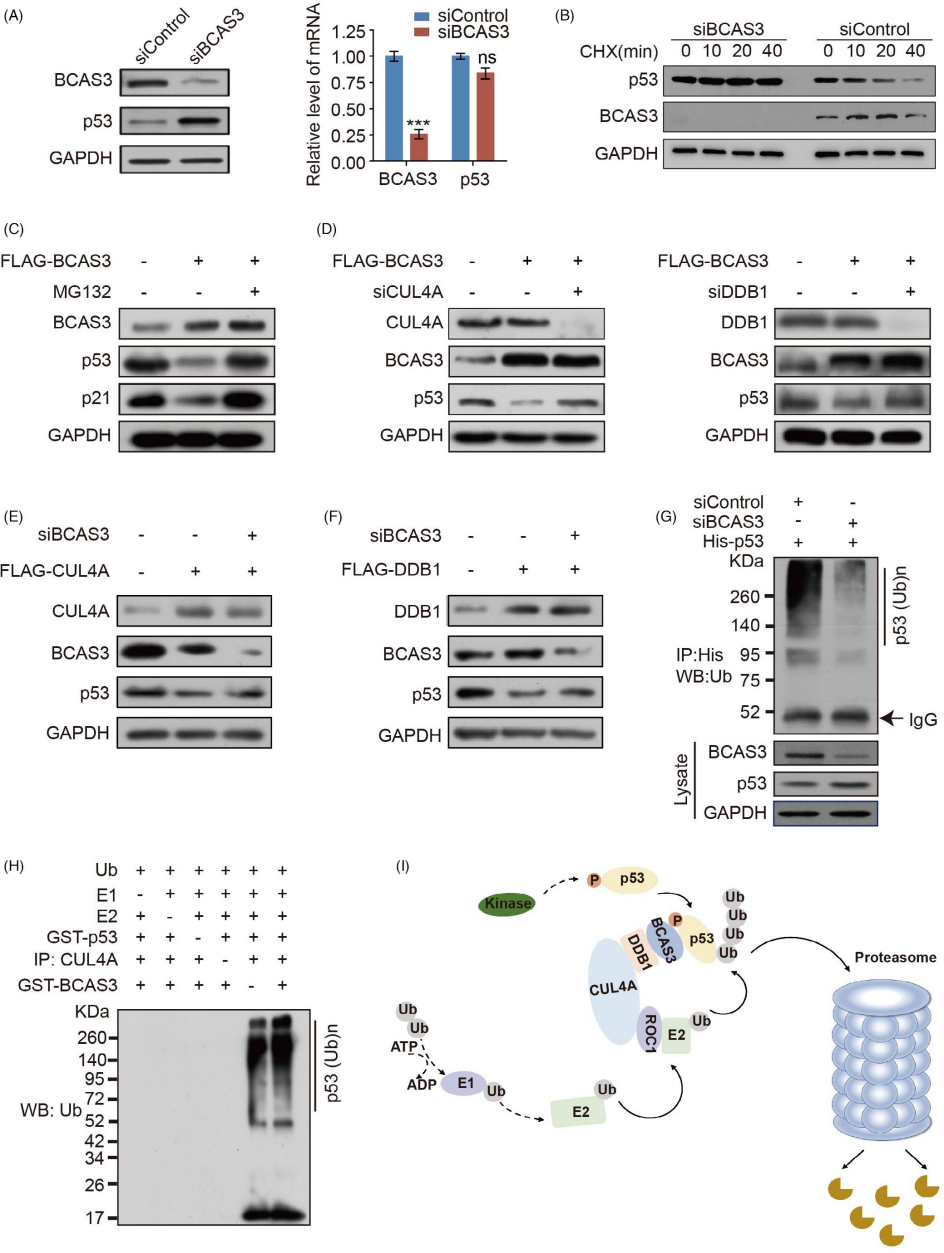
BCAS3 promotes p53 turnover via a CRL4A‐dependent mechanism. (A). MCF‐7 cells are transfected with BCAS3 siRNA, and the expression of BCAS3 and p53 are detected by Western blot analysis and RT‐qPCR. (B). BCAS3 decreases p53 half‐life. MCF‐7 cells are transfected with BCAS3 siRNA. Before harvesting, cells are treated with cycloheximide for the indicated time (CHX; 50 μg/mL). (C). BCAS3 destabilizes p53 protein. Cellular extracts are prepared from MCF‐7 cells transfected with BCAS3 and analysed by Western blot. (D). BCAS3‐associated p53 degradation is mediated by the CRL4A complex. Cellular proteins extracted from MCF‐7 cells with over‐expressing BCAS3 and knocking down CUL4A/DDB1 are subject to Western blot. (E‐F). Regulation of p53 by CUL4A/DDB1 complex is BCAS3‐dependent. MCF‐7 cell proteins over‐expressing CUL4A/DDB1 and knocking down BCAS3 are subject to Western blot. (G). In vivo p53 ubiquitination assay. MCF‐7 cells are co‐transfected with His‐p53 and BCAS3 siRNA. Cell lysates are immunoprecipitated with anti‐His antibody and immunoblotting with anti‐ubiquitin antibody. (H). In vitro ubiquitination of p53. MCF‐7 cell lysates immunoprecipitated with or without CUL4A are incubated with 20 μL reaction buffer containing purified GST‐BCAS3, GST‐p53, commercial E1, UbcH5c (E2) and ubiquitin for 1 hour at 37°C. The mixtures are subjected to Western blot with an anti‐ubiquitin antibody. (I). The predicted work model of CRL4A/BCAS3 complex for p53 degradation

To determine the potential significance that BCAS3 physically interacted with p53, we examined the role of BCAS3 on the levels of the p53 protein. BCAS3 siRNA was transfected in MCF‐7 cells, and the cellular lysates were extracted to evaluate the protein and mRNA expression levels of p53. The results indicated that p53 protein expression level was significantly increased in BCAS3 knockdown MCF‐7 cells, whereas there is no difference in terms of the level of p53 mRNA (Figure 4A). Subsequently, cycloheximide chase assays in MCF‐7 cells showed that the p53 protein half‐life was increased when BCAS3 was knocked down compared with control (Figure 4B). In accordance with these results, overexpression of BCAS3 triggered a reduction in the protein levels of p53 and p21. However, the reduction in p53 protein level related to BCAS3 can be blocked after MG132 intervention, suggesting that p53 protein degradation may occur through a proteasome‐mediated protein degradation mechanism (Figure 4C).

To examine whether the CRL4A complex is involved in BCAS3‐mediated p53 degradation, we detected p53 levels after siRNA suppression of endogenous CUL4A and DDB1 in the case of BCAS3 overexpression. The results demonstrated that knockdown of CUL4A or DDB1 diminished BCAS3‐associated p53 degradation in MCF‐7 cells (Figure 4D). We also detected the p53 level after siRNA suppression of endogenous BCAS3 in the case of CUL4A or DDB1 overexpression. The results showed that overexpression of either CUL4A or DDB1 led to the increased expression of p53, while depletion of BCAS3 reduced the expression of p53 (Figure 4E,F). Together with the above‐mentioned evidence that BCAS3 interacts with p53, these data suggest that BCAS3 is involved in regulating CRL4A‐mediated p53 degradation, and the regulation of p53 by CRL4A complex is BCAS3‐dependent.

To fully elucidate the mechanism by which BCAS3/CRL4A participates in the regulation of p53 protein degradation, we next explore whether p53 protein degradation is related to ubiquitination. MCF‐7 cells were co‐transfected with BCAS3 siRNA and His‐tagged p53. We used anti‐His antibody for immunoprecipitation and anti‐ubiquitin antibody for immunoblotting, and the result demonstrated a decreased ubiquitination level of p53 after BCAS3 knockdown (Figure 4G). In vitro ubiquitination assays showed that BCAS3 indeed enhanced the polyubiquitination of p53 when CUL4A in the reactions (lanes 5‐6), but could not promote ubiquitination in the absence of CUL4A (lanes 4‐5). (Figure 4H). Together, these data demonstrate that BCAS3 promotes p53 ubiquitination and degradation in a CRL4A‐dependent pathway (Figure 4I).

### 3.5 BCAS3‐p53 axis regulates cell growth, apoptosis and chemoresistance

**FIGURE 5:**
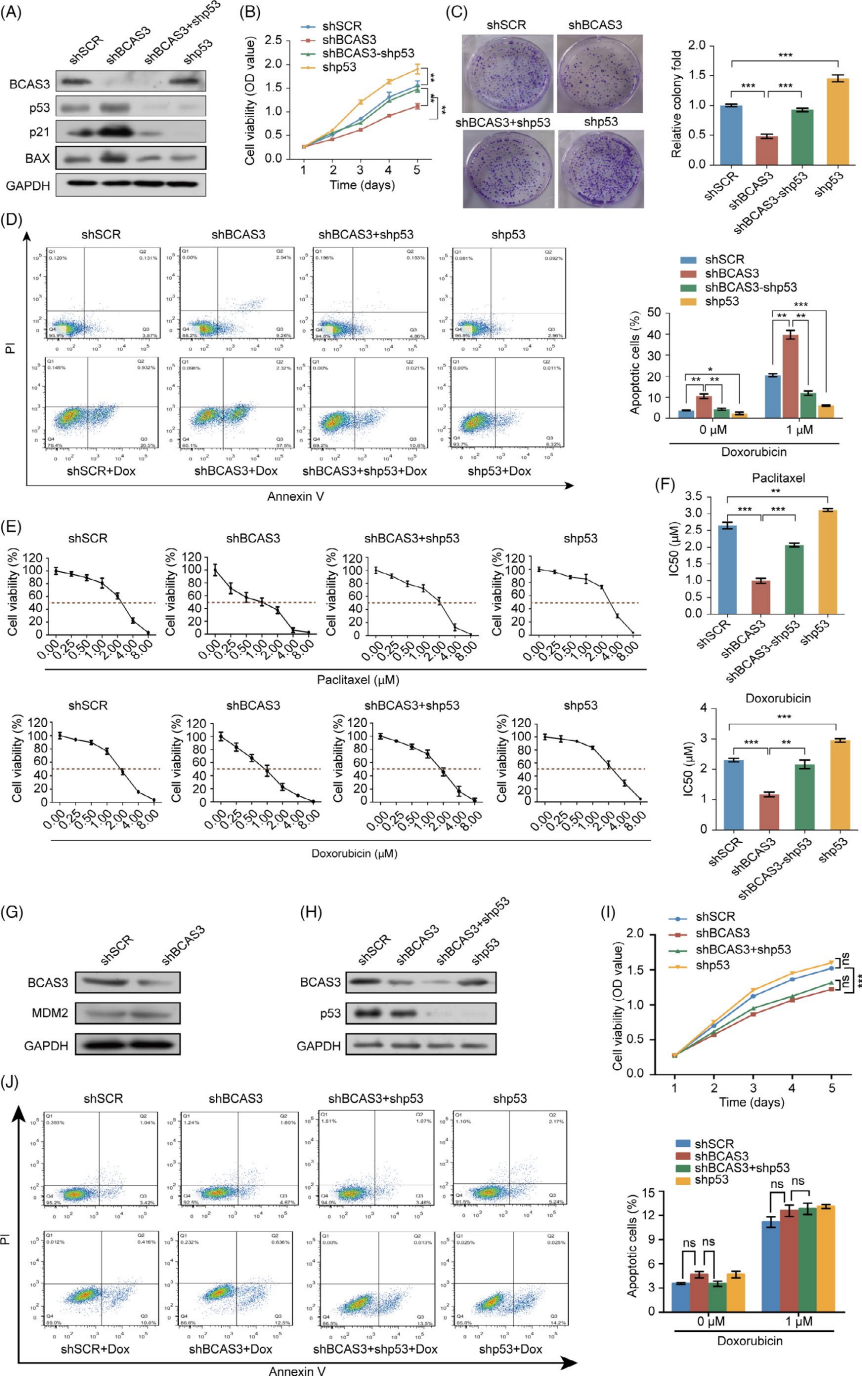
The BCAS3‐p53 axis regulates cell growth, apoptosis and chemoresistance. (A). Construction of MCF‐7 stable cell line expressing the indicated lentiviruses and Western blot analysis of p53 target genes. (B). Cell viability detection of four models. The data are presented as means ± SD of three independent experiments. (C). The relative colony fold decreases in the BCAS3 knockdown model and restores in both BCAS3 and p53 knockdown cell models. (D). Detection of apoptosis rate in four cell model. Cells are treated with or without doxorubicin for 24 hours and detected for apoptosis rate in the indicated four models (Dox; 1 μmol/L). (E). MCF‐7 stable cell lines expressing the indicated lentiviruses are treated with the indicated doses of paclitaxel or doxorubicin. (F). IC50 of paclitaxel/doxorubicin in MCF‐7 cells transfected with control shRNA, BCAS3 shRNA, p53 shRNA or both BCAS3 shRNA and p53 shRNA. The IC50 values are obtained using CCK‐8 assay. Data are presented as the mean ± SD. *P < .05, **P < .01, ***P < .001. (G). MDM2 expression upon BCAS3 knockdown and p53 stabilization. Cellular extracts from MCF‐7 cells knocking down BCAS3 are subject to examine MDM2 protein expression levels. (H). Construction of MDA‐MB‐231 stable cell line expressing the indicated lentiviruses. (I). Cell viability is detected in four cell models of MDA‐MB‐231. The data are presented as means ± SD of three independent experiments. (J). Apoptosis rate is detected in four models of MDA‐MB‐231. Cells are treated with or without doxorubicin for 24 hours and detected for apoptosis rate in the indicated four models (Dox; 1 μmol/L)

To define the physiological significance of BCAS3‐mediated destabilization of p53, we evaluated the effect of p53 on the malignant phenotype caused by BCAS3. BCAS3 was knocked down individually or in combination with p53 in MCF‐7 cells. As the target genes of p53, p21 and BAX play an important role in cell proliferation and apoptosis. Knockdown of BCAS3 increased the expression levels of these target genes, while knockdown of BCAS3 and p53 in combination could partially restore the increased levels to original status (Figure 5A). The proliferation rate of MCF‐7 cells decreased after BCAS3 knockdown, while the rate partially increased after both BCAS3 and p53 knockdown (Figure 5B,C). Treated with doxorubicin for 24 hours, the results revealed that the cell apoptosis rate of the BCAS3 knockdown group (37.5%) was increased compared with control (20.5%); however, this effect could be partially reversed via p53 knockdown in combination (10.8%). A similar trend can be seen in the absence of doxorubicin (Figure 5D).

Since kinds of literature had confirmed that p53 plays an irreplaceable role in the chemoresistance of breast carcinoma, our aforementioned results demonstrated that BCAS3 promoted p53 protein ubiquitination degradation; therefore, we speculated that chemoresistance conferred by BCAS3 may be mediated by p53. MCF‐7 cells were treated with paclitaxel/doxorubicin after overexpression or knockdown of BCAS3, and the dose‐response curves of four groups were shown (Figure 5E). Compared with the IC50 in shSCR group (paclitaxel, 2.75 µmol/L; doxorubicin 2.33 µmol/L), the IC50 in shBCAS3 cells (paclitaxel, 0.99 μmol/L; doxorubicin 1.19 µmol/L) was decreased, while inhibition of p53 expression could partially increase the IC50 (paclitaxel, 2.14 µmol/L; doxorubicin 2.22 µmol/L). (Figure 5F). In comparison with MCF‐7 shp53 cells, MCF‐7 shBCAS3‐shp53 cells have less BCAS3 proteins, and more p53 proteins theoretically. Although the Western blot results showed that MCF‐7 shBCAS3‐shp53 cells have the same little p53 protein levels as MCF‐7 shp53 cells, they had more p21 protein levels. As a downstream of p53, p21 played inhibitory functions in cell viability, colony formation and chemoresistance. So, shBCAS3‐shp53 versus shp53 showed a significant difference in cell viability, colony formation as well as chemoresistance. In addition, BCAS3 may promote cell viability, colony formation and chemoresistance in p53‐independent pathways.

To define other pathways that are involved in p53 protein homeostasis, we detected the expression of MDM2 with BCAS3 knockdown in MCF‐7 cells. Our results revealed that BCAS3 knockdown could not affect the protein levels of MDM2 (Figure 5G). We propose that regulating p53 protein homeostasis by BCAS3 is MDM2‐independent.

In addition, we choose p53‐mutated MDA‐MB‐231 cells, which had no wild‐type p53 activity or non‐functional p53 expression.^33^, ^34^, ^35^, ^36^ BCAS3 was knocked down individually or in combination with p53 in MDA‐MB‐231 cells (Figure 5H). The proliferation rate of MDA‐MB‐231 cells decreased after BCAS3 knockdown, while the rate did not show an upward trend after both BCAS3 and p53 knockdown (Figure 5I). Treated with doxorubicin for 24 hours, the cell apoptosis rate of the BCAS3 knockdown group (12.5%) partially increased compared with the control (10.6%), while no statistical difference was observed between the two groups. In addition, this effect could not be partially reversed via p53 knockdown in combination (13.5%). A similar trend can be seen in the absence of doxorubicin (Figure 5J). The results indicate that the p53‐dependent function of BCAS3 in regulating proliferation and apoptosis is partially blocked in MDA‐MB‐231 cells.

Overall, the above results demonstrate that the BCAS3‐p53 axis is involved in biological functions, including cell proliferation, apoptosis and chemoresistance.

### 3.6 High expression of BCAS3 is correlated with low levels of p53 in nuclear and is an independent parameter for predicting poor prognosis of patients with BRCA

**FIGURE 6:**
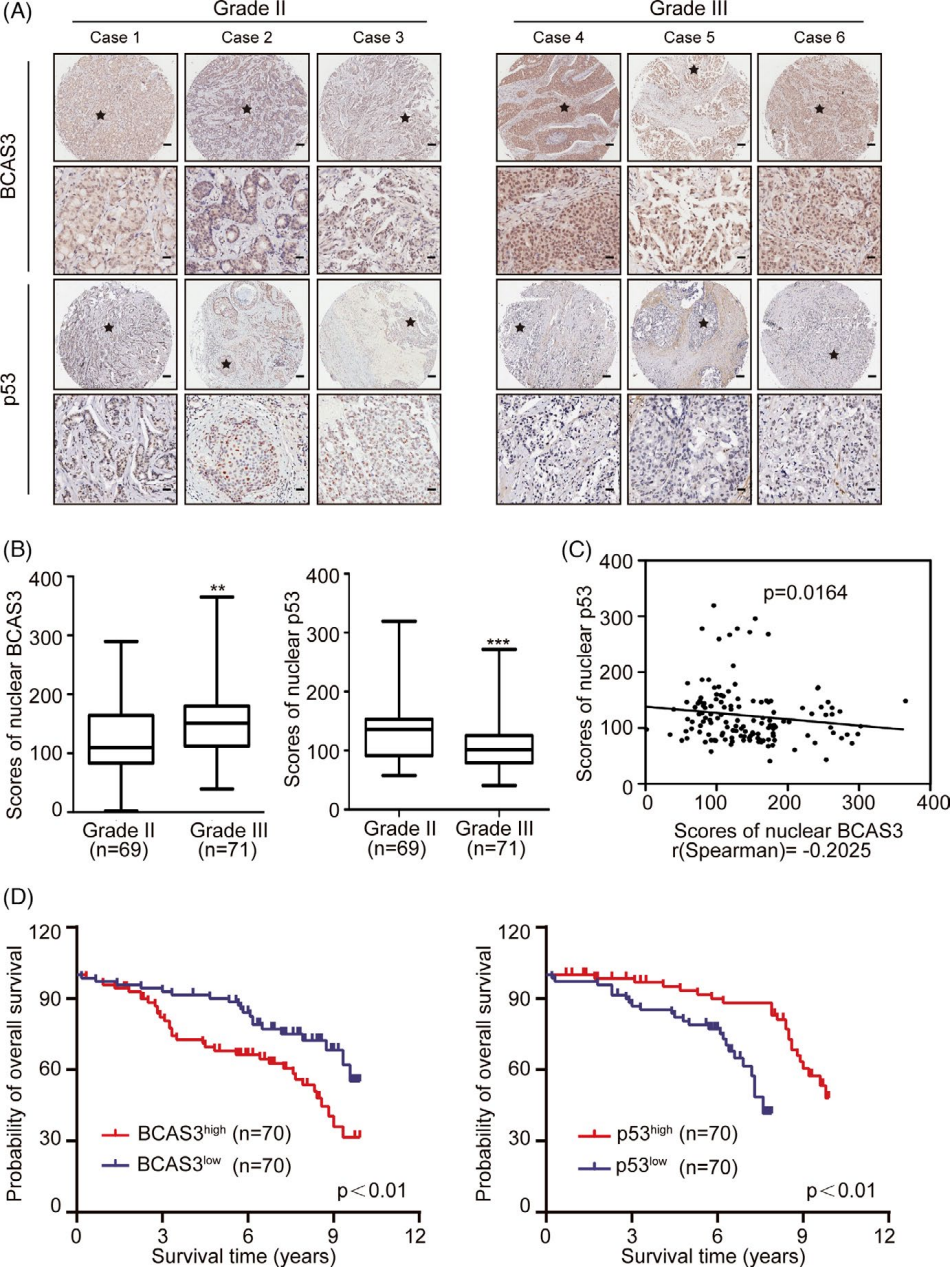
BCAS3 and/or p53 correlates with poor prognosis. (A). Immunohistochemical staining of BCAS3 and p53 in tissue arrays containing 140 breast tumour samples (grade II: 69, grade III: 71). Representative image immunostaining with anti‐BCAS3 and anti‐p53 is presented. Scale Bars, 100 µm for ‘up’ and 25 µm for ‘down’. (B). Histoscores for BCAS3 and p53 staining in different grades. (C) Correlation between BCAS3 and p53 (n = 140, Spearman's correlation coefficient *R* and *P*‐value are shown). (D). Kaplan‐Meier curves of the OS in patients with BRCA. *P < .05, **P < .01, ***P < .001

To further explore the clinical value of BCAS3 and p53, we examined the expression of p53 in tissues from a cohort of 140 patients with BRCA using semiquantitative IHC analysis. The results demonstrated that BCAS3 and p53 were both present in the nuclei of breast cancer cells. Tumour cells displayed high expression levels of BCAS3 (63.4%, 101 of 140) and low expression levels of p53 (74.7%, 108 of 140), respectively. The level of nuclear BCAS3 was positively correlated with histological grades, while a negative correlation was found between nuclear p53 levels and histological grades of the breast carcinoma samples (Figure 6A,B). Additionally, we found that BCAS3 expression was negatively correlated with p53 expression using semiquantitative IHC (*r* = −0.1718, *P* = .0432; Figure 6C). Kaplan‐Meier curves showed that patients with high levels of BCAS3 were more likely to have decreased survival rate, and an opposite prognostic trend was observed in p53 expression (*P* < .01) (Figure 6D).

The relationship between expression levels of BCAS3 and the clinicopathological characteristics of 140 patients with BRCA was investigated, as listed in Table 1. The results demonstrated that BCAS3 expression was associated with histological grade (*P* = .004), tumour size (*P* = .004) and TNM stage (*P* < .001). Multivariate analysis showed that BCAS3 expression was an independent predictor of overall survival (HR = 11.979, *P* < .001; Table 2). These results indicate that BCAS3 is an independent parameter for the poor prognosis of patients with breast tumours.

**TABLE 1:** Correlation between clinicopathologic characteristics and BCAS3 expression

Variables	NO. of patients	Nuclear BCAS3 level		*P*‐value
		BCAS3^low^ (%)	BCAS3^high^ (%)	
Total cases	140	69 (49.3)	71 (50.7)	
Age (y)				
≥55	82	41 (50.0)	41 (50.0)	0.865
<55	58	28 (48.3)	30 (51.7)	
Location				
Left	62	26 (41.9)	36 (58.1)	0.13
Right	78	43 (55.1)	35 (44.9)	
Tumour size (cm)				
D ≤ 3.3	82	49 (59.8)	33 (40.2)	**0.004**
D > 3.3	58	20 (34.5)	38 (65.5)	
Oestrogen receptor				
Positive	83	43 (51.8)	40 (48.2)	0.496
Negative	57	26 (45.6)	31 (54.4)	
Progesterone receptor				
Positive	56	33 (58.9)	23 (41.1)	0.084
Negative	84	36 (42.9)	48 (57.1)	
HER2				
Positive	134	66 (49.3)	68 (50.7)	0.646
Negative	6	3(86.5)	3 (13.5)	
Histological grade				
Ⅱ	69	43 (62.3)	26 (37.7)	**0.004**
Ⅲ	71	26 (36.6)	45 (63.4)	
TNM stage				
Ⅰ	21	17 (81.0)	4 (19.0)	**<0.001**
Ⅱ	79	41 (51.9)	38 (48.1)	
Ⅲ	39	11 (28.2)	28 (71.8)	

*P* < 0.05 was considered statistically significant. Pearson's chi‐square test was used.

**TABLE 2:** Univariate and Multivariate analysis of factors associated with OS

Variables	Univariate analysis			Multivariate analysis		
	HR	95% CI	*P*	HR	95% CI	*P*
BCAS3^high^ vs BCAS3^low^ expression	12.827	4.577‐35.948	**<.001**	11.979	4.132‐34.725	**<.001**
Age	0.755	0.414‐1.375	.358	0.740	0.387‐1.414	.362
Location	0.817	0.449‐1.489	.510	0.927	0.481‐1.789	.822
Tumour size	1.828	1.003‐3.331	.**049**	1.294	0.674‐2.483	.438
Oestrogen receptor	0.766	0.420‐1.399	.387	0.785	0.354‐1.738	.550
Progesterone receptor	0.567	2.296‐1.088	.088	0.740	0.316‐1.732	.488
HER2	1.959	0.270‐14.239	.506	1.496	1.980‐11.334	.697
Grade II vs Grade III	1.180	0.648‐2.150	.588	0.573	0.294‐1.115	.101
stage I vs stage II vs stage III	2.100	1.286‐3.428	.**003**	1.308	0.793‐2.159	.293

Abbreviations: 95%CI, 95% confidence interval; OS, overall survival.

*P* <0.05 was considered statistically significant.

## 4 DISCUSSION

DNA amplification is frequently observed in several chromosomal sites allowing tumour development and progression, as well as conferring drug resistance. A previous study demonstrated that 17q23 is a common region of amplification, occurring in approximately 20% of primary breast tumours.^37^ BCAS3 was found to have an amplification rate of 9.4% in primary breast tumours. Furthermore, BCAS3 was reported to be rearranged and fused to BCAS4 which was located at 20q13. The chromosomal site also tended to amplify in breast cancer. The BCAS3 and BCAS4 fusion was detected in the MCF‐7 cell line and led to high levels of mRNA expression.^5^ However, the function of BCAS3 in breast cancer has not yet been defined and needs to be explored experimentally. In this study, we found that BCAS3 promoted tumour cell properties linked to tumorigenesis through degrading p53 and explained the functions of BCAS3 in amplification and fusion in breast carcinoma. Compared with normal samples, we found that BCAS3 expression was remarkably elevated in breast cancer tissues. Moreover, we determined that a high level of BCAS3 positively correlated with prognosis in a cohort of 1071 patients from LinkedOmics, which was in accord with the evidence from a previous study.^38^ Therefore, we speculate that high BCAS3 expression may be related to tumour malignancy.

Subsequently, we explored the biological functions of BCAS3 in MCF‐7 cells, and the results demonstrated that high BCAS3 expression could promote growth, which was in accord with a previous study.^39^ The proliferation rate of tumour cells is determined by the balance between proliferation and apoptosis. Further experiments revealed that BCAS3 overexpression inhibited apoptosis. It has been reported that BCAS3 excessive expression in premenopausal breast tumours seems to weaken the therapeutic effect of tamoxifen.^40^ As the MCF‐7 cell line belongs to the ER + breast cancer subtype based on data from the ATCC, we determined whether BCAS3 affected chemoresistance. The results showed that BCAS3 overexpression partially reduced the sensitivity of MCF‐7 cells to paclitaxel and doxorubicin. Taken together, BCAS3 increased the number of MCF‐7 cells by elevating the proliferation rate and conferring chemotherapy resistance.

Coping response to cellular stress and carcinogenic signals, p53 regulates the cell cycle to maintain cell's steady state, and its level is mainly regulated by ubiquitination modification.^41^ The CRL4A complex is composed of CUL4A, DDB1 and ROC1.^16^ The cullin subunit of CUL4A acts as a scaffold and bridges the substrate through one end, while the other end is responsible for ROC1 connection to recruit E2. In addition, several studies^16^, ^42^, ^43^ demonstrated that inactivation of CUL4A results in an increase in p53 and downstream genes in both human and mouse cells, indicating that the CRL4A complex may be involved in p53 expression regulation. Furthermore, CUL4A remained amplified in breast carcinomas,^44^ and these data may indicate that dynamic changes in the expression of the CRL4A complex contribute to breast oncogenic properties.

Mechanistically, the substrate recruiting of CRL4A complex implies that DDB1, which either directly interacts^27^, ^28^ or associates with WD40 repeat adaptor proteins, binding to the substrate protein.^29^, ^30^, ^31^ In this study, we showed that BCAS3, a novel WD40 repeat‐containing protein, regulates p53 protein stability during ubiquitin‐dependent proteolysis. As CUL4A was previously found to interact with p53, bridging p53 to other E3 ligases to promote its proteasomal degradation,^31^ we sought to verify whether BCAS3 acts as an adaptor and binds to p53 and CRL4A complex. Our study further determined that BCAS3 directly interacted with both DDB1 and p53 as substrate‐specific adaptors, suggesting that BCAS3 acts as a mediator for CRL4A ubiquitination of substrate proteins.

Many E3 ubiquitin ligases, including MDM2, TRIM3, TRIM 28 and TRIM 45, are involved in p53 ubiquitination and p53 protein homeostasis. MDM2‐mediated ubiquitination represents a key mechanism driving proteasomal degradation of p53.^45^ MDMX is a structural homolog of MDM2, but it does not harbour intrinsic E3 ubiquitin ligase activity. MDMX plays an essential role in p53 degradation in vivo by recruiting UbcH5c to facilitate MDM2 E3 ligase function.^46^ Many factors and proteins can target the MDM2‐p53 axis to regulate p53 protein levels. For example, RLIM is a downstream target of TRIM28 and functions between TRIM28 and MDM2 and thereby acts as an MDM2 inhibitor to regulate p53 protein levels.^47^ TRIM45 could promote K63‐linked polyubiquitination and inhibit K48‐linked polyubiquitination of p53 by MDM2. In our results, no interaction between BCAS3 and MDM2 was illustrated by immunopurification‐mass spectrometric analysis.^48^ Knockdown of BCAS3 in MCF‐7 cells also did not affect the protein levels of MDM2. Therefore, we did not pay much attention to the effect of BCAS3 on MDM2‐mediated p53 degradation. In the future, we will pay more attention to whether other pathways that are involved in p53 protein homeostasis might be affected by BCAS3.

Given that BCAS3 can stabilize p53, it is not unexpected to speculate that BCAS3 could regulate cell proliferation, apoptosis and chemoresistance in a p53‐dependent manner. Clinical evidence from ER‐positive tumours showed that most samples with TP53 wild type (WT) were resistant to chemotherapy, while ER‐negative subtypes are more sensitive to chemotherapy due to TP53 mutations.^49^ Subsequently, several reports revealed that tumours with TP53 WT rarely achieved a complete response to chemotherapy. It may be due to the induction of senescence after doxorubicin treatment, while lack of growth capture in mutant tumours can lead to cell death and clinical outcome.^50^, ^51^ In recent years, there have been many studies on the chemoresistance of MCF‐7, which is a breast cancer cell line expressing wild‐type p53 protein. For example, a vital role of SET protein was identified in paclitaxel‐induced chemoresistance in MCF‐7 cells.^52^ Elevated integrin α5β1 can promote doxorubicin resistance in MCF‐7 cells by downregulating ERK protein kinase.^53^ In this study, we showed that knockdown of BCAS3 conferred chemosensitivity to MCF‐7 cells by increasing p53 protein levels. Taken together, these data provide insights into therapy for TP53 WT breast cancers through overexpression of p53 in cancer cells.

Our study provided solid evidence that BCAS3 overexpression exerted vital functions in breast cancer progression via post‐translational inactivation of p53. Mechanistically, we showed that BCAS3 interacted with the CRL4A complex and promoted the degradation of p53 protein by ubiquitin‐dependent proteolysis. IHC results from a cohort of patients with BRCA showed that both BCAS3 and p53 were localized in the nuclei of breast cancer cells. Clinically, patients with BRCA with elevated BCAS3 and decreased p53 levels displayed a poorer prognosis.

## 5 CONCLUSIONS

We demonstrated that BCAS3 promoted growth and inhibited apoptosis of breast tumour cells by participating in the regulation of p53 ubiquitination mediated by the CRL4A complex. Further, BCAS3 may be a novel predictor for prognosis and guide chemotherapy regimens for patients with breast cancer.

## CONFLICTS OF INTEREST

None.

## AUTHOR CONTRIBUTIONS

Z Zhou, Y Yang and Y Wang designed this study. Z Zhou and RF Qiu performed experiments and collected the data. W Liu searched the database and analysed the data. Z Zhou, Y Yang and HF Yu analysed the data. Z Zhou, TS Yang and G Li prepared the figures. W Huang, X Teng and YK Yang advised on this manuscript. Z Zhou drafted the manuscript. Y Yang, HF Yu and Y Wang reviewed and revised the manuscript. All authors read and approved the final manuscript for publication.

## Supporting information

Supplementary Material

## Data Availability

All data generated or analysed during this study are included in this article.
