# Supplementary material for: BCAS3 exhibits oncogenic properties by promoting CRL4A‐mediated ubiquitination of p53 in breast cancer
Source: Cell Prolif. 2021 Jul 9;54(8):e13088. doi: 10.1111/cpr.13088 (PMC8349660; doi:10.1111/cpr.13088)
Supplement: Supplementary file 1 — Supplementary Material [file CPR-54-e13088-s001.docx]

**Supporting information**

**Reagents and plasmid**

The reagents applied in this study were as follows: MG132 (Sigma–Aldrich, USA, 20 µM), doxorubicin (MCE, China), paclitaxel (MCE, China), cycloheximide (Sigma–Aldrich, USA, 20 µg/mL) FLAG-ubiquitin (Sigma–Aldrich, USA), E1 (Boston Biochem, USA), E2 (UbcH5c, Boston Biochem, USA). For Co-IP assay and cell transfection, full-length BCAS3, CUL4A, DDB1, and p53 were cloned into pCMV-Tag 2B, or pcDNA3.1-Myc-His (-) C Vector as indicated. For the GST pull-down assay, BCAS3, DDB1, p53 and the truncates were cloned into a pGEX-4T-3 expression vector.

**Real-time RT-PCR**

TRIzol reagent (Invitrogen, USA) was used to extract total RNA. A total of 2 µg RNA) was used to synthesize cDNA using the Reverse Transcription kit (Roche, Germany). Relative quantitation was performed by measuring real-time SYBR Green fluorescence and analyzed using 2^-ΔΔCt^ method. GAPDH was used as a control. Real-time primer sequences are shown in *Supporting Table S3.*

**Lentiviral infection and gene silencing**

The recombinant lentivirus for BCAS3 overexpression (ovBCAS3) and BCAS3-knockdown lentivirus (shBCAS3) were purchased from GenePharma (Shanghai, China). The target sequences of the shRNAs are listed in *Supplementary Table S4*. To establish stable cell lines, MCF-7 cells were infected with lentivirus for 24 h. siRNAs against human BCAS3 and the non-targeting control were purchased from GenePharma (Shanghai, China). Transient transfection of siRNAs was performed using Lipofectamine 2000 (Invitrogen, USA). The siRNA sequences are listed in *Supporting Table S4.*

**Tissue Microarrays and Immunohistochemistry (IHC)**

Human breast tissue arrays of 140 cancer samples (*HBreD140Su07*) and 40 matched adjacent normal breast tissues (*HBreDu046Sur02*) were obtained from Shanghai Outdo Biotech. The clinicopathological information was defined as previously described^1^, and follow-up data were collected at the end of July of 2014, with a median follow-up of 61months (range: 4-67 months). IHC was performed according to a standard protocol^2^ with anti-BCAS3 antibody and anti-p53 antibody. The staining results were assessed by two independent observers blinded to both the clinical characteristics and the sample. BCAS3 expression was evaluated according to H-scores. In brief, H-score was comprehensively evaluated based on staining intensity and the percentage of staining area. staining intensity was classified as 0 (negative), 1 (weak), 2 (moderate) and 3 (strong). A reaction for BCAS3/p53 was scored based on the sum obtained by multiplying the intensity grade by the percentage of staining area. Then, we employed ROC analysis to determine the cut-off values of BCAS3^high^/BCAS3^low^ and p53^high^/p53^low^ for tumor “positivity” by using the 0, 1-criterion.

**Supporting Table S1. Mass Spectrometry analysis of BCAS3-containing protein complex**

| Band | Identified proteins | Peptides |
| --- | --- | --- |
| 332kDa | DSP | RLNDSILQATEQRR  KAITGFDDPFSGKT |
| 73kDa | KHSRP | RSVSLTGAPESVQKA  RIGGGIDVPVPRH |
| 119kDa | KIF11 | KNILNKPEVNQKL  KLLNTVEETTKD  KIGAVEEELNRV  KAVDQHNAEAQDIFGKN  RVITALVERT  KNGVYISEENFRV  RTTAATLMNAYSSRS  KFCADSDGFSQELRN  REAGNINQSLLTLGRV |
| 70kDa | HSP70 | KITITNDKGRL  KVEIIANDQGNRT  RTTPSYVAFTDTERL  KNALESYAFNM*KS  KDAGVIAGLNVLRI |
| 51kDa | BAG5 | RLQEIQKEVKS  KQLLALDAVDPQGEEKC |
| 54kDa | Vimentin | RLGDLYEEEMRE  RLQDEIQNMKEEMARH  KNLQEAEEWYKS  RVEVERDNLAEDIMRL  RQVQSLTCEVDALKG  K.M*ALDIEIATYR.K  KLQEEMLQREEAENTLQSFRQ  REM*EENFAVEAANYQDTIGR.L  REMEENFAVEAANYQDTIGRL  RKVESLQEEIAFLKK  KILLAELEQLKG |
| 53kDa | p53 | KSVTCTYSPALNKM  RLGFLHSGTAKS |
| 50kDa | PTP1B | RIQNAGGSVM*IQR.V  RIQNAGGSVMIQRV  RSGSTAVGVMISPKH  RNVIEAVYSRL |
| 36kDa | PP2A | RMAGDPVANVRF  RNLCSDDTPMVRR |
| 52kDa | SBP1 | KLNPNFLVDFGKE  KGGFVLLDGETFEVKG |
| 72kDa | hnRNP F | KHSGPNSADSANDGFVRL  RQSGEAFVELGSEDDVKM |
| 48kDa | Ruvbl2 | RKGTEVQVDDIKRV  RAVLIAGQPGTGKT  RQASQGMVGQLAARR  KDKVQAGDVITIDKA  RLLIVSTTPYSEKD  RGLGLDDALEPRQ  KTTEMETIYDLGTKM  RAAGVVLEMIRE  RVYSLFLDESRS |
| 50kDa | Ruvbl1 | KQAASGLVGQENARE  RAVLLAGPPGTGKT  KGLGLDESGLAKQ  KTALALAIAQELGSKV |
| 72kDa | PRMT5 | RVPLVAPEDLRDDIIENAPTTHTEEYSGEEKT  RDLNCVPEIADTLGAVAKQ |

**Supporting Table S2. The primary antibodies for western blotting, IP and IHC**

| Antibody | Concentration | | | | Specificity | Company | Catalog # |
| --- | --- | --- | --- | --- | --- | --- | --- |
|  | WB | IHC | | IP |  |  |  |
| BCAS3 | 1:2000 | | 1:200 | 2ug/mL | Rabbit polyclonal | Abcam | ab71162 |
| CUL4A | 1:5000 | | / | 2ug/mL | Rabbit monoclonal | Abcam | ab92554 |
| p53 | 1:1000 | | 1:200 | 2ug/mL | mouse monoclonal | Santa Cruz | sc-126 |
| p21 | 1:2000 | | / | / | Rabbit monoclonal | Abcam | ab109520 |
| ubiquitin | 1:1000 | | / | / | mouse monoclonal | Cell Signaling Technology | 3936 |
| GAPDH | 1:5000 | | / | / | Mouse monoclonal | Proteintech | HRP-60008 |
| DDB1 | 1:50000 | | / | 2ug/mL | Rabbit monoclonal | Abcam | ab109027 |
| ROC1 | 1:2000 | |  | 2ug/mL | Rabbit monoclonal | Abcam | ab2977 |
| MDM2 | 1:1000 | | / | / | Rabbit polyclonal | proteintech | 19058-1-AP |
| BAX | 1:1000 | | / | / | Mouse monoclonal | proteintech | CL488-60267 |
| His-tag |  | |  | 2ug/mL | Mouse monoclonal | Santa Cruz | Sc-8036 |

**Abbreviations:** WB, western blot; IP, immunoprecipitation; IHC, immunohistochemistry

**Supporting Table S3. Primer sequences for real-time PCR (5’-3’)**

| Primers for real-time PCR | | |
| --- | --- | --- |
| BCAS3 | forward | CATCATCACAGTTATTGACACC |
|  | reverse | AGATGTCCTGTACTTTGGCT |
| CUL4A | forward | ACCTCGCACAGATGTACCAG |
|  | reverse | AGGTTGACGAACCGCTCATTC |
| ROC1 | forward | TTGTGGTTGATAACTGTGCCAT |
|  | reverse | GACGCCTGGTTAGCTTGACAT |
| DDB1 | forward | ATGTCGTACAACTACGTGGTAAC |
|  | reverse | CGAAGTAAAGTGTCCGGTCAC |
| p21 | forward | GATGGAACTTCGACTTTGTCAC |
|  | reverse | GTCCACATGGTCTTCCTCTG |
| p53 | forward | CAGCACATGACGGAGGTTGT |
|  | reverse | TCATCCAAATACTCCACACGC |
| GAPDH | forward | GGAGCGAGATCCCTCCAAAAT |
|  | reverse | GGCTGTTGTCATACTTCTCATGG |

**Supporting Table S4. Sequences for siRNA and shRNA (5’-3’)**

| **Sequences for siRNA of BCAS3** | | |
| --- | --- | --- |
| siBCAS3-1 | forward | GCACCUUAGUGGAACACAUTT |
|  | reverse | AUGUGUUCCACUAAGGUGCTT |
| siBCAS3-2 | forward | GCAGAUGCAGUACAGUAUUTT |
|  | reverse | AAUACUGUACUGCAUCUGCTT |
| siBCAS3-2 | forward | GCGGCAGCAUACCAAGAAATT |
|  | reverse | UUUCUUGGUAUGCUGCCGCTT |
| **Target sequences for shRNA on BCAS3** | | |
| Target 1 | CTGCAGATGCAGTACAGTATT | |
| Target 2 | ACAGTTATCTCATCCAGTT | |
| **Target sequences for shRNA on p53** | | |
| Target | GAAATTTGCGTGTGGAGTA | |

Reference:

1. Wang H, Wu J, Meng X, et al. MicroRNA-342 inhibits colorectal cancer cell proliferation and invasion by directly targeting DNA methyltransferase 1. *Carcinogenesis.* 2011;32(7):1033-1042.

2. Haigis MC, Sinclair DA. Mammalian sirtuins: biological insights and disease relevance. *Annu Rev Pathol.* 2010;5:253-295.
